# Precision Medicine and the Human Proteome in Disease, Diagnostics and Translation: Current Status and Future Prospects

**DOI:** 10.3390/biomedicines13051130

**Published:** 2025-05-07

**Authors:** M. Walid Qoronfleh

**Affiliations:** 1 Healthcare Research & Policy Division, Q3 Research Institute (QRI), 7227 Rachel Drive, Ypsilanti, MI 48917, USA; walidq@yahoo.com; Tel.: +1-(734)-664-8368; 2 Healix Lab, Al Khuwair South, Muscat 123, Oman; 3 AtomGen, Esenşehir Mah. Güneyli Sokak No:15/1, 34776 Ümraniye, İstanbul, Türkiye

**Keywords:** human proteome, proteomics, multi-omics, proteins, biomarkers, precision medicine, artificial intelligence, AI, mass spectrometry

## Abstract

The human proteome—the entire collection of proteins expressed by the human genome—represents a dynamic and intricate landscape of biological function. Proteins are the workhorses of the body, driving processes from cellular communication to immune defense, and their alterations underpin many diseases. Understanding the proteome has become a cornerstone of modern biomedical research, offering insights into disease mechanisms, diagnostic tools, and personalized treatments through precision medicine. This commentary explores the current state of human proteome research; its applications in disease understanding, diagnostics, and therapeutic advancements; and the exciting prospects that lie ahead.

## 1. Introduction: The Human Proteome and Its Importance

The human genome contains approximately 20,000 protein-coding genes, yet the proteome is vastly more complex. Processes like alternative splicing, where a single gene produces multiple protein variants, and post-translational modifications, such as phosphorylation or glycosylation, generate a diverse array of proteins known as proteoforms. Estimates suggest that the human proteome may comprise hundreds of thousands of distinct protein species, far exceeding the number of genes.

The Human Proteome Project (HPP), launched in 2010 by the Human Proteome Organization (HUPO), aims to map this complexity by identifying and characterizing all proteins encoded by the human genome [HUPO]. By 2024 [1], the HPP has detected evidence for approximately 93% of predicted human proteins, a testament to the field’s rapid progress. This effort relies on advanced technologies like mass spectrometry and antibody-based profiling, with data cataloged in resources such as UniProtKB [2] and the Human Protein Atlas [3].

*Why does the proteome matter?* Proteins are not static entities; their expression, structure, and interactions shift in response to physiological states, including disease. This makes the proteome a critical lens for the following:

**Understanding disease**: proteins are often the effectors of disease processes. In cancer, for instance, mutated proteins drive uncontrolled cell growth, while in neurodegenerative disorders like Alzheimer’s, misfolded proteins accumulate and disrupt brain function.

**Diagnostics**: disease-specific proteins, or biomarkers, can be detected in blood, urine, or tissue, enabling early diagnosis and monitoring. For example, elevated levels of prostate-specific antigen (PSA) signal prostate cancer risk.

**Precision medicine**: by analyzing an individual’s proteome, clinicians can tailor treatments to a patient’s unique molecular profile like oncogenes/oncoproteins analysis, thus, enhancing efficacy and reducing side effects.

Despite these advances, challenges remain. The remaining 7% of the proteome—termed “missing proteins”—are elusive due to their low abundance, tissue-specific expression, or transient nature. Overcoming these hurdles is key to unlocking the proteome’s full potential. This perspective highlights the current status of proteome research, its practical applications, and the future directions that promise to transform medicine.

## 2. Current Status and Applications

*Mapping the Human Proteome: A Milestone in Progress*

As of 2024, the HPP reports that 18,138 of the 19,411 predicted protein-coding genes (based on the GENCODE reference [4]; as classified by neXtProt—no longer provides data or services—it is 19,823 predicted protein-coding genes; note: neXtProt is replaced by UniProt & HPP Portal, a knowledge hub to support the HPP Grand Challenge) have been credibly detected at the protein level, achieving 93% coverage. This milestone reflects over a decade of collaborative effort, integrating data from mass spectrometry, antibody validation, and bioinformatics/computational biology. Initially, the HPP has operated through two strategic initiatives:

**Chromosome-Centric HPP (C-HPP)**: focuses on mapping proteins encoded by each chromosome, ensuring comprehensive coverage.

**Biology/Disease-Driven HPP (B/D-HPP)**: targets proteins relevant to specific biological processes or diseases, such as cardiovascular, cancer and inflammation.

With continued community efforts, the focus is now turned onto understanding the proteome in its entirety with the formation of the “HPP Grand Project—A Function for Every Protein”. The mission for this grand project is to understand the proteins’ function, biological significance and network/interactomes.

The following table summarizes the HPP progress (Table 1).

Mass spectrometry is considered the workhorse of proteomics and the backbone of the HPP effort. It separates and identifies proteins based on their mass-to-charge ratio. Complementary tools, like the Human Protein Atlas’s antibody-based imaging resource, map protein expression across tissues, revealing spatial and functional diversity or ProteomeXchange data resource [5] are essential tools to advance human proteome research. Yet, the missing 7%—often proteins expressed in rare cell types or under specific conditions—pose a technical challenge requiring continued innovative approaches and technology development. Development of cutting-edge proteomics high-throughput technologies like Aptamer-based or Multiplex fluorescent-labeled bead-detection, Proximity extension assay (PEA), protein microarrays (forward-phase or reverse-phase), Biochips (e.g., multiplexed ELISA platform) are a few examples [6], also including other non-mass-spectrometric methods [7].

*The Proteome in Disease Research*

Proteomics has transformed our understanding of disease by revealing the protein players in pathogenesis. By comparing proteomic profiles between healthy and diseased states, researchers identify biomarkers, therapeutic targets, and molecular pathways [8]. Below are some key examples.

**Cancer**: proteomics uncovers proteins driving malignancy. In breast cancer, overexpression of human epidermal growth factor receptor 2 (HER2) is a well-known marker and target for Trastuzumab^®^ therapy. Proteomic studies also reveal tumor heterogeneity, showing how protein expression varies within a single cancer, guiding personalized treatment.

**Neurodegenerative diseases**: in Alzheimer’s, proteomics quantifies tau and amyloid-beta proteins in brain tissue and cerebrospinal fluid (CSF), linking their accumulation to cognitive decline. This has deepened our understanding of disease progression and highlighted potential therapeutic targets.

**Infectious diseases**: during the COVID-19 pandemic, proteomic analysis of infected cells identified host proteins hijacked by SARS-CoV-2, such as those involved in inflammation, offering clues for drug development.

A striking case comes from colorectal cancer research. A 2023 study used mass spectrometry to profile tumor tissues, identifying proteins linked to metastasis, such as integrins and matrix metalloproteinases. These findings suggest new biomarkers for early detection and therapeutic targets to halt cancer spread [9].

Techniques driving these discoveries include the following:

**Liquid Chromatography-Tandem Mass Spectrometry (LC-MS/MS)**: detects thousands of proteins in a sample, providing a global proteomic snapshot.

**Protein microarrays**: screen for protein interactions or expression changes in a high throughput manner.

**AI and bioinformatics**: analyzes complex datasets to pinpoint disease-relevant proteins or signatures.

*Proteomics in Diagnostics*

Diagnostics is a flagship application of proteomics, leveraging proteins as disease indicators. Biomarkers—proteins uniquely associated with a condition—enable early detection, prognosis, and treatment monitoring. Proteomics excels here due to its ability to detect proteins in accessible fluids like blood or urine [10].

**Established biomarkers**: PSA for prostate cancer and troponins for heart attacks are proteomic success stories. These proteins, detectable via simple blood tests, guide clinical decisions.

**Emerging biomarkers**: in ovarian cancer, a proteomic signature in blood has shown promise for early detection, with sensitivity and specificity exceeding traditional markers like CA-125. Similarly, plasma proteomics in cardiovascular disease predicts risk and monitors progression.

**Tools and technologies**: mass spectrometry offers unparalleled sensitivity, detecting proteins at picomolar levels. ELISA targets specific proteins, while protein chips enable rapid, multiplexed analysis.

A compelling example is Alzheimer’s diagnostics. A 2022 study used mass spectrometry to analyze cerebrospinal fluid, identifying a protein panel—including tau isoforms and neurofilament light chain—that distinguished Alzheimer’s from other dementias with over 90% accuracy. Such tests could shift diagnosis to earlier, more treatable stages [11].

Challenges include translating these findings into routine clinical use. Biomarkers must be validated across diverse populations, and assays must be cost-effective and reproducible. Nevertheless, proteomics is poised to expand the diagnostic toolkit significantly [10].

*Translation into Precision Medicine*

Precision medicine tailors’ treatments to individual patients, and proteomics is a linchpin in this revolution. By revealing a patient’s molecular profile, proteomics informs treatment selection and predicts outcomes [12,13].

**Patient stratification**: in cancer, proteomic data classifies tumors beyond genetic mutations. For instance, high PD-L1 (programmed death-ligand 1) expression identifies patients likely to benefit from immunotherapy drugs like Pembrolizumab^®^.

**Pharmacoproteomics**: this field examines how proteins influence drug responses. In leukemia, proteomic analysis of drug-resistant cells has revealed proteins mediating resistance, guiding alternative therapies.

**Multi-omics integration**: combining proteomics with genomics and transcriptomics provides a holistic view of disease. A 2023 colon cancer study integrated these datasets, improving survival predictions and identifying patients suited for adjuvant chemotherapy.

A practical example is breast cancer management. Proteomic profiling of tumors distinguishes hormone receptor-positive cases (responsive to tamoxifen) from triple-negative cases (requiring aggressive chemotherapy), reducing overtreatment and optimizing outcomes.

The promise of precision medicine lies in its specificity. By matching therapies to proteomic signatures, clinicians can avoid trial-and-error approaches, enhancing efficacy and minimizing side effects. However, integrating proteomic data into clinical workflows requires robust infrastructure and standardized protocols.

## 3. Future Prospects: Emerging Technologies and Challenges

The human proteome’s potential is vast, and emerging technologies are set to accelerate its exploration and application [6,7].

*Advancements on the Horizon*

Some of the noteworthy breakthroughs are the following.

**Single-cell proteomics**: traditional proteomics averages protein expression across cell populations, masking variability. Single-cell proteomics, enabled by advances in mass spectrometry sensitivity, analyzes individual cells, revealing heterogeneity in diseases like cancer. This could refine diagnostics and uncover rare cell populations driving disease.

**Artificial Intelligence (AI) and machine learning**: proteomic datasets are massive and complex. AI tools can identify patterns, predict disease trajectories, and suggest novel biomarkers. For example, AI-driven analysis of mass spectrometry data has improved protein identification accuracy by 20% in recent studies.

**Proteoform analysis**: current efforts focus on detecting proteins, but cataloging proteoforms—variants with distinct modifications—offers deeper insights. New techniques, like top-down mass spectrometry, are beginning to map this diversity, potentially revealing disease-specific signatures.

Completing the proteome map could yield transformative discoveries. For rare diseases, where single protein defects often dominate, a full catalog might pinpoint elusive targets. In common conditions like diabetes, proteomic insights could reveal new pathways for intervention.

*Challenges Ahead*

Despite this promise, hurdles remain [10].

**Technical barriers**: low-abundance proteins, such as those in plasma masked by dominant proteins like albumin, are hard to detect. Enrichment strategies and next-generation instruments are needed.

**Data standardization**: variability in proteomic methods across labs complicates data sharing and validation. The HPP’s guidelines aim to unify standards; nevertheless, adoption is incomplete.

**Clinical translation**: moving from discovery to bedside requires large-scale validation studies, regulatory approval, and cost-effective assays. However, this process can take years, delaying patient benefits.

A 2024 HPP report [1] estimates that completing the proteome map could take another decade, but the payoff—new diagnostics, therapies, and a deeper understanding of biology—justifies the effort.

*Vision and Opportunities*

The future of proteomics is not just about completion, but also application. Imagine a world where the following are true.

A blood test profiles your proteome to predict disease risk years in advance.

Cancer treatments are selected based on a tumor’s real-time proteomic signature, monitored and updated during therapy.

Rare disease patients receive diagnoses and therapies based on their unique protein defects.

Undoubtedly, achieving this vision requires collaboration across academia, industry, and healthcare entities, alongside sustained investment in research, technology, and professional training.

## 4. First Edition Coverage

Taking into consideration the discussion above, the first edition of human proteome/proteomics collection offers an insightful group of papers. Xie et al. provide an in-depth review of the bridge between the human proteome discoveries into precision medicine. On the other hand, Long et al., Anirudhan et al., and Naja et al. explore the pharmacoproteomic of the human proteome in diabetes and cancer, while Tonry et al., Urbiola-Salvador et al. and Tucci et al. delve into biomarker discovery and proteomic profile characterization of diseases like heart failure, COVID-19, and chronic pelvic pain, respectively. These types of studies reveal disease-relevant pathways and promising biomarker candidates or signatures that potentially could represent valuable targets for paving the way for the differential diagnosis and therapeutic management. Further, Kononikhin et al. and Núñez et al. present quantitative proteomics methodology/workflow development for the plasma proteome a pivotal sample source in the context of precision medicine for its unique insight into the health and disease status of individuals. Finally, McDowell et al. demonstrate the value of biosampling techniques, sample collection devices, and sample preparation protocols, which are a critical step in proteomics analysis and proteome investigation. Here, they show a lateral flow device is a viable blood separation and transportation tool for untargeted and targeted proteomics applications.

## 5. Conclusions

The human proteome stands at the nexus of biology and medicine, offering a window into health and disease. With 93% of predicted proteins detected by 2024, the HPP has laid a strong foundation, driving advances in disease research, diagnostics, and precision medicine. Current applications—uncovering cancer biomarkers, refining Alzheimer’s diagnostics, tailoring cancer therapies—demonstrate its impact. Looking forward, innovations like single-cell proteomics and AI promise to push boundaries further, though challenges in detection, standardization, and translation persist.

As the proteome map nears completion, its integration into clinical practice could redefine healthcare, delivering precise, personalized solutions. The journey from 93% to 100% is more than a scientific milestone—it is a pathway to better patient care and lives.

## Figures and Tables

**Table 1:** Translating the code of life. Current status of the human proteome project (HPP) [1].

Metric	Value	Notes
Predicted proteins (GENCODE 2024) Predicted proteins (neXtProt 2023)	19,411 19,778	Based on latest genomic annotations Based on latest release/HUPO report
Detected proteins (protein evidence-PE1, 2024 and 2023)	18,138 18,397	93% of predicted proteins confirmed
Missing proteins (PE2-4, 2024) Missing proteins (PE2-4, 2023)	1273 1381	Low-abundance or tissue-specific proteins
Percent proteome discovered	93%	Calculated as (18,138/19,411) or (18,397/19,778) × 100 = 93%

## Data Availability

This is a literature-based opinion article. No new data were created or analyzed in this study. Data sharing is not applicable to this article.
